# CGRP Antagonism and Ketogenic Diet in the Treatment of Migraine

**DOI:** 10.3390/medicina60010163

**Published:** 2024-01-15

**Authors:** Francesca Finelli, Alessia Catalano, Michele De Lisa, Giuseppe Andrea Ferraro, Sabino Genovese, Federica Giuzio, Rosanna Salvia, Carmen Scieuzo, Maria Stefania Sinicropi, Fabiano Svolacchia, Antonio Vassallo, Alessandro Santarsiere, Carmela Saturnino

**Affiliations:** 1 U.O.C. Pediatrics -S. Giuseppe Moscati Hospital, 83100 Avellino, Italy; medfinelli@gmail.com; 2 Department of Pharmacy-Drug Sciences, University of Bari “Aldo Moro”, 70126 Bari, Italy; alessia.catalano@uniba.it; 3 U.O.C. Hygiene, Epidemiology and Public Health Department ASP, 85100 Potenza, Italy; michele.delisa@aspbasilicata.it; 4 Plastic and Reconstructive Surgery Unit, Multidisciplinary Department of Medical-Surgical and Dental Specialties, University of Campania Luigi Vanvitelli, 80138 Naples, Italy; giuseppe.ferraro@unicampania.it; 5 Department of Agriculture, Faculty of Viticulture and Oenology, Federico II University, 83100 Avellino, Italy; sabinogenovese1@gmail.com; 6 Department of Sciences, University of Basilicata, 85100 Potenza, Italy; r.salvia@unibas.it (R.S.); carmen.scieuzo@unibas.it (C.S.); antonio.vassallo@unibas.it (A.V.); alessandro.santarsiere@unibas.it (A.S.); carmela.saturnino@unibas.it (C.S.); 7 U.O.C. Primary Care and Territorial Health, Social and Health Department, State Hospital, 47893 San Marino, San Marino; 8 Spinoff TNCKILLERS s.r.l., University of Basilicata, 85100 Potenza, Italy; 9 Spinoff XFlies s.r.l., University of Basilicata, 85100 Potenza, Italy; 10 Department of Pharmacy and Health and Nutrition Sciences, University of Calabria, 87036 Arcavacata di Rende, Italy; s.sinicropi@unical.it; 11 Department of Sense Organs, La Sapienza University, 00185 Rome, Italy; fabiano.svolacchia@gmail.com

**Keywords:** migraine, CGRP, gepants, headache, ketogenic diet

## Abstract

The study of migraine is based on the complexity of the pathology, both at the pathophysiological and epidemiological levels. Although it affects more than a billion people worldwide, it is often underestimated and underreported by patients. Migraine must not be confused with a simple headache; it is a serious and disabling disease that causes considerable limitations in the daily life of afflicted people, including social, work, and emotional effects. Therefore, it causes a daily state of suffering and discomfort. It is important to point out that this pathology not only has a decisive impact on the quality of life of those who suffer from it but also on their families and, more generally, on society as a whole. The clinical picture of migraine is complex, with debilitating unilateral or bilateral head pain, and is often associated with characteristic symptoms such as nausea, vomiting, photophobia, and phonophobia. Hormonal, environmental, psychological, dietary, or other factors can trigger it. The present review focuses on the analysis of the physiopathological and pharmacological aspects of migraine, up to the correct dietary approach, with specific nutritional interventions aimed at modulating the symptoms. Based on the symptoms that the patient experiences, targeted and specific therapy is chosen to reduce the frequency and severity of migraine attacks. Specifically, the role of calcitonin gene-related peptide (CGRP) in the pathogenesis of migraine is analyzed, along with the drugs that effectively target the corresponding receptor. Particularly, CGRP receptor antagonists (gepants) are very effective drugs in the treatment of migraine, given their high diffusion in the brain. Moreover, following a ketogenic diet for only one or two months has been demonstrated to reduce migraine attacks. In this review, we highlight the diverse facets of migraine, from its physiopathological and pharmacological aspects to prevention and therapy.

## 1. Introduction

Migraine is a chronic neurological disease that occurs during adolescence but can also affect children, with a lower prevalence in people over the age of 50. It is a specific type of very complex headache that affects 6–8% of men and 12–14% of women in the world [1]. According to the World Health Organization (WHO), migraine ranks seventh among the most disabling diseases in the world and third when considering only female sex. Several studies have been conducted on the prevention and treatment of migraine [2,3] by exploring new goals [4]. Migraine attacks can last for a long period of time, from 4 h to several days, and are often associated with symptoms such as vomiting, nausea, and high sensitivity to light and noise. Paleness, diarrhea, fatigue, and difficulty concentrating can be further side effects. Gender and age play a predominant role in the onset of migraine [5,6]. The most affected people are between the ages of 15 and 45, with a prevalence in women. In women, migraine attacks are more disabling, longer, and have more associated symptoms. In migraine sufferers, the cells of the nervous system are more sensitive to stimuli, which alters their normal balance. In this way, various electrical impulses are generated and spread through the brain causing the various symptoms that precede the attack, such as difficulty in speaking, loss of control of movements, and blurred vision. Of particular importance is the involvement of the trigeminal nerve [7]. Stimulation of this nerve causes the release of modulators involved in the inflammatory response causing pain in the membranes that line the brain and in the blood vessels. There are no treatments that can eliminate migraines, but drug treatments can reduce the pain, severity, and frequency of attacks. In most of the affected population, migraines recur periodically for a maximum of 15 days per month. If these levels are exceeded, the migraine becomes chronic. With advancing age, migraine attacks become sporadic with reduced intensity until they completely disappear [8]. In trying to reduce both the frequency of attacks and their duration, it is crucial to identify the factors that can trigger a migraine. They can be enclosed in five macro-groups: (1) Hormonal factors: These include the rise or fall of normal hormone levels in women. Girls suffer from migraines to a greater extent than boys due to the high levels of the hormone estrogen, which peaks during pregnancy, in the period before and during the menstrual cycle, and to a lesser extent, during menopause. (2) Dietary factors: Foods and beverages such as chocolate, alcohol, coffee, sausages, soy products, smoked fish, nuts, and foods containing glutamate and tyramine (e.g., potato chips, yogurt, and bananas) can trigger migraine. (3) Environmental factors: Migraine can also be triggered by changes in weather or excessive sensory stimulation, for example, flashing lights, strong smells, and loud noises. (4) Psychological factors: Stress, anxiety, and changes in mood can play a role in migraine onset. (5) Other factors: These include alterations in the interactions of the brain stem with the trigeminal nerve, alterations of electrolyte balances in the brain, in particular of the neurotransmitter serotonin, little sleep or, sometimes, too much sleep, particularly strong and intense physical effort, the use and/or abuse of particular drugs, high blood sugar levels, and metabolic disorders. Additionally, individuals who already have migraine sufferers in their families will be more likely to experience the disease. The first classification of the various forms of headache was conducted by the committee of experts of the International Headache Society (IHS) in 1988, which made it possible to identify and catalog the different forms of headache using a common terminology. The latest edition, “International Classification of Headache Disorders (ICHD-3rd edition beta version, called ICHD-3)”, was published in 2018 and is included in the ICD-11, the *International Classification of Diseases* published by the WHO [9,10]. It is described in these recent papers [11,12]. Recently, some types of headaches, including medication overuse headaches, new daily persistent headaches, and persistent refractory headaches [13], have been attributed to severe acute respiratory syndrome coronavirus 2 (SARS-CoV2) infection, the virus responsible for coronavirus disease-19 (COVID-19) [14]. In addition, headache, specifically migraine, is among the most frequent symptoms persisting or newly developing as part of so-called long COVID or post-COVID syndrome [15,16]. From several studies conducted on the pathogenesis of migraine, it has emerged that one of the most important aspects of migraine is the genetic component, which determines the appearance of the disease [17]. In this review, various types of migraine are considered, focusing on the therapies used for this disease, specifically the use of CGRP antagonist and the importance of a balanced diet, as well as physical activity.

## 2. Classification of Headaches

The different headaches are classified using a hierarchical scale, with an increasing level of diagnostic accuracy. A detailed prognosis requires analysis of all five levels to acquire more information; however, in common practice, only first- or second-level diagnoses are commonly applied. More than 300 types of headache are distinguished and grouped into 14 headache categories [18]. The first four classes identify primary headaches, which occur when the headache does not depend on other pathologies, whereas secondary headaches belonging to the 5th to the 12th groups occur in close relationship with and are caused by another pathology that can have serious consequences. The 13th and 14th groups include cranial neuralgia, facial pain, and other types of headaches. Primary headaches are divided into tension-type headaches [19], migraines, cluster headaches [20], and other primary headaches [21]. Secondary headaches can occur in relation to various problems, including head trauma, vascular or nonvascular cranial disorder, use or withdrawal of a substance, presence of infections, alterations in normal hormonal and metabolic balances, psychiatric disorders, and skull-related disorders related to the neck (whiplash), face, teeth, ears, and nose.

## 3. Calcitonin Gene-Related Peptide (CGRP) Receptor

**Figure 1:**
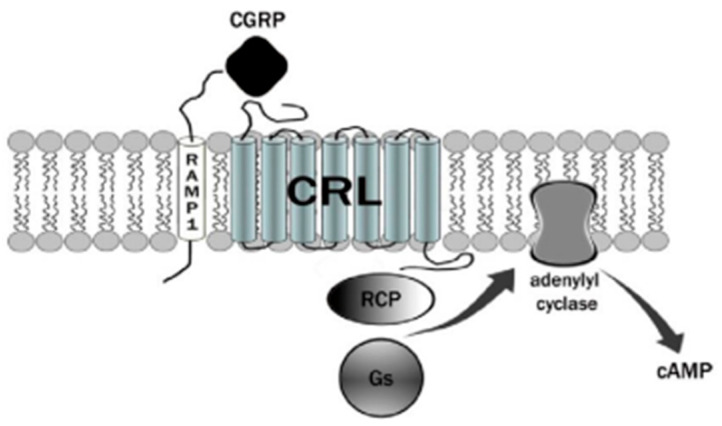
The CGRP receptor is a member of the B family of G-protein-coupled receptors (GPCR); calcitonin receptor-like receptor (CRL); receptor component protein (RCP); stimulatory G protein (Gs).

The CGRP receptor is a member of the B family of G-protein-coupled receptors (GPCRs) (Figure 1) [22]. This receptor is characterized by seven transmembrane helices, an N-terminal extracellular domain, and a C-terminal intracellular domain. It is divided into three main parts: RAMP1 (receptor activity modifying protein 1), a small transmembrane protein; CRL (calcitonin receptor-like receptor); and a cytoplasmic protein required for signal transduction, namely RCP (receptor component protein). The extracellular domain RAMP1 is critical in the binding of CGRP receptor antagonists [23]. In addition to RAMP1, there are two other proteins, RAMP2 and RAMP3, that share the same transmembrane structure of 22 amino acids and the intracellular C-terminal portion of nine residues. The N-terminal extracellular domain is different and consists of about 90 residues for RAMP1 and RAMP3 and 103 residues for RAMP2. RAMPs are abundantly diffused within our organism and distributed in almost all tissues. RAMP1 is expressed in the heart, uterus, brain, bladder, and pancreas and the skeletal, muscular, and gastrointestinal systems. High expression of RAMP2 has been demonstrated in the lung, heart, placenta, skeleton, muscles, and pancreas, whereas RAMP3 is widely expressed in humans. The CRL receptor is a seven-transmembrane receptor capable of interacting specifically with each of the RAMP proteins, which confers selectivity to the ligand. CRL with RAMP 1 gives rise to a CGRP receptor, whereas CRL with RAMP2 forms an AM1 receptor and RAMP3 forms AM2. The AM1 and AM2 receptors are part of the family of adrenomedullin (AM) receptors, a peptide belonging to the CGRP family along with the calcitonin (CT) and amylin (AMY) peptides. The third component is constituted by the RCP protein necessary in some biological functions, such as the association of the receptor with cellular metabolic pathways [24,25].

The binding mechanism by which CGRP binds to the receptor is represented by the two-domain model developed by Hoare [26]. According to this model, a first affinity trap is formed by the interaction of the C-terminal domain of CGRP with the extracellular N-terminal domain of both CRl and RAMP1 [27]. This binding causes an increase in the concentration of the peptide, which allows the N-terminal portion of the CGRP to connect with the juxtamembrane portion of the CRL, resulting in the activation of the receptor with a consequent increase in cAMP. The increase in cAMP is due to the presence of the receptor-associated G protein. Normally, a stimulatory G protein called Gs is capable of activating adenylate cyclase, which in turn activates cAMP-dependent protein kinase A. Nerve endings containing CGRP are widespread from the adventitial to the medial layer of blood vessels. The increase in CGRP together with the increase in cAMP leads to one of the effects responsible for migraine pain, vasodilation. Vasodilation is caused by a direct relaxation mechanism of the smooth muscle cells from the vessels and increased by a second NO-dependent cellular mechanism. In the first mechanism, a direct link between the CGRP peptide and its receptor, both through the direct release of the peptide and by diffusion, is recorded. In the second, the synthesis of NO by the enzyme NO synthase (NOS) activates the guanylate cyclase with subsequent production of cGMP and vasodilation. NO is able to upregulate CGRP in trigeminal ganglion neurons [28]. The molecular mechanisms of migraine by NOS and neuropeptides have been recently reviewed [29]. CGRP and its receptors are expressed in the trigeminal ganglion, as well as in the afferent nerve endings that transmit sensory stimuli in the periphery and in the endings of the caudal trigeminal. The presence of nerve endings containing CGRP receptors and their central activity demonstrates the key role in migraine pathogenesis. Interestingly, CGRP has also been shown to inhibit NO production in vascular endothelial cells [30].

## 4. Therapies Based on the Different Forms of Migraine

Different forms of migraine exist, ranging from those with mild symptoms, such as fatigue and sensitivity, changes in sounds, or slight muscle tension, to those with high pain levels that cause sufferers to cease any activity in progress to retreat to a dark and silent environment to regain mental clarity. This is one of the reasons why migraine is considered a disabling disease that impairs normal daily work, home, and leisure activities. Preventive treatment can improve the quality of migraine attacks and decrease their frequency [31]. The choice of preventive drug is based on the patient’s medical history, i.e., the presence of any other problems, other taken drugs, and the side effects of the drug to be administered. The latter is one of the reasons to choose to prescribe a drug that, in addition to preventing migraines, treats the patient’s other pathologies. For example, in a patient suffering from heart problems, the most suitable drugs are beta-blockers, such as propranolol and topiramate, which are used in the treatment of epilepsy and are an excellent therapy for migraine prophylaxis, and amitriptyline, which is used in antidepressant therapy for insomnia (Table 1) [32]. The choice of drugs varies according to the intended purpose. In the case of a mild migraine, the medications used are pain relievers, including FANS (ibuprofen, acetylsalicylic acid, or acetaminophen) and antiemetic drugs such as metoclopramide to relieve both vomiting and nausea, as well as the 5imegepae itself. However, none of these oral treatments, including beta-blockers, triptans, antiepileptic drugs, and tricyclic antidepressant drugs, were developed for migraines, and they are only able to reduce the frequency of migraine attacks by 50% in a small percentage of patients. For severe migraines, on the other hand, in addition to the administration of antiemetic drugs, triptan drugs are used concomitantly with intravenous fluids to compensate for any loss caused by vomiting [33]. The use of indomethacin has been indicated for refractory COVID or post-COVID headaches, as well as common analgesics, anti-inflammatory drugs, and/or triptans [34,35]. To relieve typical symptoms, such as throbbing pain, drugs that can stop migraine at its onset are used. For this purpose, ditans can be used; these have similar pharmacodynamics to triptans but have greater efficacy and tolerability. Gepants are also able to stop migraines; they block the release of CGRP, which is capable of causing migraines, and together with ditans, they are part of a new therapy used for the treatment of the disease [36]. A class of drugs that has prevailed in migraine treatment in recent years is anti-CGRP monoclonal antibodies [37,38]. These are administered through subcutaneous injection and target CGRP, blocking its advancement. However, these drugs should not be taken by cardiopathic and hypertensive patients [39]. Pain relievers, such as nonsteroidal anti-inflammatory drugs (NSAIDs) or acetaminophen, are used to treat mild to moderate migraines and can be taken alone or in combination with triptans. Overuse of analgesics can both worsen the severity of each attack and increase its frequency. More specifically, abuse occurs when a drug is taken for more than two weeks per month for a period exceeding three months. In fact, these forms of headaches are known as drug abuse headaches. When these drugs all fail to have any useful pharmacological effects, opioid analgesics are considered as a last resort. Moreover, three innovative strategies are described for the treatment of migraine: one linked to the role of the CGRP, another linked to nutritional strategies and innovative diet therapy protocols such as the ketogenic diet, and transcutaneous electrical nerve stimulation (TENS) therapy, which is a fast-acting, effective therapy for the treatment of acute migraine that is used in emergency departments [40,41]. TENS is a noninvasive analgesic technique used in the treatment of nociceptive, neuropathic, and musculoskeletal pain [42,43].

### 4.1. CGRP Antagonists

The first antagonist drug to be developed was olcegepant, administered intravenously due to its large volume. Telcagepant was subsequently introduced to create an orally bioavailable drug, which is very effective in the acute treatment of migraine. Developing new antagonists is difficult due to the polymorphic nature of the receptor. Elevated levels of CGRP can be associated with other conditions, such as sepsis and hypertension, and ocular conditions, such as trauma. The antagonists of the CGRP receptor are very effective drugs in the treatment of migraine given their high diffusion in the brain. CGRP is a neuropeptide made up of 37 amino acids spread both in the central and peripheral nervous systems. The use of CGRP antagonists has increased since the 1980s because it has been discovered to alleviate both pain and the symptoms associated with migraine. The peptide is present in the meninges, nuclei of the brainstem, and cortex, as well as the ganglial and cervical levels. When given to patients suffering from migraines, CGRP antagonists can cause migraine-like headaches [44]. It reaches higher concentrations during the migraine attack, causing vasodilation with a consequent increase in pain transmission that decreases after the administration of acute treatments. In the first phase of migraine, neurons release substance P, CGRP, and mast cells, which are neuropeptides with vasodilating effects and molecules with inflammatory properties, respectively. The result is the dilation of the intracranial and extracranial vessels, which become more permeable, releasing proinflammatory substances in the meninges and giving rise to the typical pulsatility of pain. It is demonstrated, therefore, that migraine pain is actually pain of the meninges and not of the brain itself. These processes lead to the first sensitization of peripheral nociceptors (primary sensitization), which is involved in the transduction of noxious stimuli into signals. These signals, represented by the release of CGRP and glutamate by the trigeminal at the central level, reach the caudal trigeminal nucleus located in the brain stem, cross the thalamus, and arrive at the somatosensory cortex, where the signal is converted into conscious sensation [45,46]. The antinociceptive system becomes less efficient due to a dysfunction of the serotonergic and noradrenergic nuclei. For this reason, drugs that restore normal levels of serotonin and noradrenaline are used. Opiates, stress, and physical exertion are other possible contributors to the release of CGRP. Moreover, exposure to KCl and capsaicin causes migraines by activating the inflammatory system and increasing the release of CGRP [47]. Activation of CGRP receptors leads to increased levels of cyclic AMP (cAMP), which modulates the intracellular activity of calcium-dependent kinase enzymes such as protein kinase A (PKA) and C (PKC). PKA and PKC are involved in the phosphorylation and subsequent activation of glutamate receptors such as 8imegep Gluk6 and NMDA/NR1. Subsequently, the phosphorylation of the NR1 subunit leads to the release of magnesium, which stimulates the excitatory postsynaptic potential (EPSP), causing an increased influx of ions. This stimulation of the postsynaptic potential ensures the continuation of cortical depression in migraine with aura and without aura [48]. Molecules such as olcegepant and telcagepant prevent binding of the initial CGRP, blocking the entire receptor activation process [49,50,51]. New oral drugs for migraine [52,53,54] include atogepant, 8imegepant, and 8imegepant [55,56], which have been recently reviewed [57].

### 4.2. Monoclonal Antibodies

Monoclonal antibodies have led to a breakthrough, demonstrating their efficacy and safety even in long-term treatments. These antibodies exhibit a rapid onset of effect. They can quickly provide the intended treatment benefits, even in patients who have not responded to previous preventive treatments or are concurrently using oral preventive treatments. An antibody is an immunoglobulin produced by plasma cells, which are a component of the immune system involved in the body’s defense mechanisms. The antibodies have a particular Y-shaped structure and are able to recognize specific antigens, i.e., any molecule capable of binding to immunoglobulin. An antigen can be a protein, lipid, phospholipid or complex formed between a drug and an endogenous molecule. In this case, the antibody binds CGRP, blocking its release centrally, thus relieving pain in migraine sufferers. There are five different types of immunoglobulins: IgA, IgG, IgD, IgM, and IgE, which differ in structure and molecular weight. The immunoglobulins present in the greatest concentration are of the IgG type, which is in turn divided into four subclasses (IgG1, IgG2, IgG3, and IgG4). Their administration is monthly, or in some cases quarterly, through subcutaneous or intramuscular intravenous injection. Four monoclonal antibodies are used in the treatment of migraine: erenumab [58], fremanezumab [59], galcanezumab [60,61,62], and eptinezumab [63,64]. In general, a therapy is effective when at least one of the following conditions occurs: a 50% reduction in the frequency of days in which headaches occur; a significant decrease in the severity of the attacks; a significant decrease in the duration of the attacks; a reduction of migraine-related disability; and an improvement in health-related quality of life.

### 4.3. Innovative Medicines

Recent studies have been especially focused on medicine that can be considered “innovative”, including considering arthropods as a source of innovative molecules to complement traditional medicine. Particular interest has been shown in the venom of arthropods (snakes, spiders, insects, etc.), a complex mixture composed of different proteins, peptides, and compounds that have not yet been well identified [65,66]. Some compounds, or the in toto mixture, may have vasoconstrictor [67,68] or anti-inflammatory [69,70,71] effects, which could help to reduce the effect of the migraine.

## 5. Recent Preclinical and Clinical Studies on Gepans and Monoclonal Antibodies

Killoran et al. (2023) [72] reported a study on novel CGRP receptor antagonists for migraine therapy that involved improving the antagonist potency of the known peptide (^34^Pro,^35^Phe)CGRP_27–37_ through the structural modification of truncated C-terminal CGRP peptides. Six peptide analogues were synthesized and evaluated in both in vitro and in vivo studies. Results from in vitro studies showed that a peptide containing 10 amino acids (P006) was the key candidate to be tested for in vivo evaluation. This peptide also demonstrated antagonistic activity in vivo after intraperitoneal injection into mice. P006 was formulated as a preparation suitable for nasal administration by spray-drying with chitosan to form mucoadhesive microcarriers (9.55 ± 0.91 mm diameter) and loading 0.2 mg of peptide per 20 mg dose. Greco et al. (2022) [73] studied the interaction of CGRP with other pain mediators relevant for neuronal sensitization in an animal model of chronic migraine by using the formalin test. The results of this study showed that the activation of the CGRP pathway in a migraine animal model was associated with the persistent activation of inflammatory pathways, which was paralleled by a condition of hyperalgesia. Specifically, male Sprague-Dawley rats were used for the assay. They were exposed to nitroglycerin (NTG, 5 mg/kg, i.p.) or vehicle coadministered with the CGRP receptor antagonist olcegepant (2 mg/kg i.p.) or its vehicle every other day over a 9-day period. One day after the last injection of NTG (or vehicle), a behavioral test and ex vivo analysis were performed. Olcegepant was shown to reduce NTG-induced trigeminal hyperalgesia in the second phase of the orofacial formalin test. Moreover, it reduced gene expression and protein levels of CGRP, proinflammatory cytokines, inflammatory-associated miRNAs (miR-155-5p, miR-382-5p, and miR-34a-5p), and transient receptor potential ankyrin channels in the medulla–pons area, cervical spinal cord, and trigeminal ganglia. It also reduced the NTG-induced increase in CGRP and inflammatory cytokines in serum. Haghdoost et al. (2023) [74] and Messina et al. (2023) [75] recently reported a systematic review and network meta-analysis of phase three randomized controlled trials evaluating the efficacy, safety, and tolerability of CGRP monoclonal antibodies and gepants for the preventive treatment of migraine. Specifically, studies on anti-calcitonin gene-related peptide monoclonal antibodies (erenumab, eptinezumab, fremanezumab, or galcanezumab) and gepants (atogepant, 9imegepant) were included in analysis in 19 studies on 14,584 participants with episodic and/or chronic migraine. All interventions, except for eptinzumab 30 mg, considerably lowered mean monthly migraine days in comparison to placebo. All medications showed a responder rate ≥50% than placebo, and the results were statistically significant in patients receiving the drugs subcutaneously or intravenously, but not orally. All medications considerably lowered mean monthly headache days, although no data supported these results for 9imegepant and eptinezumab. The systematic reviews and network meta-analysis protocols were developed following the Preferred Reporting Items for Systematic Reviews and Meta-analyses (PRISMA) guidelines and registered in the PROSPERO database (registration number: CRD42022310579).

## 6. Ketogenic Diet

The role of the ketogenic diet for the management of symptoms and pathology has been increasingly defined over the years. The ketogenic diet was developed in the 1920s for the treatment of epilepsy in children; however, in recent years, it has also been used for the treatment of various neurological diseases with increasing success. In recent years, the use of some diets for the treatment of migraine has also been investigated [76,77,78]. The ketogenic diet exploits particular physiological processes that are activated only under certain conditions: during a prolonged fast or when the quantity of sugars introduced with food is very low. In both cases, the stores of glycogen, a form of accumulation of sugars, in the liver and tissues are almost exhausted. In this situation, most organs and tissues switch to using fatty acids as a source of energy, except for the brain, red blood cells, and type II muscle fibers, which are unable to exploit this substrate. The liver, using fatty acids as raw material, begins to produce ketone bodies—acetone, acetoacetate, and β-hydroxybutyric acid—which become the primary fuel to keep the most sensitive organs and tissues functioning, in particular the brain. The increase in ketone body concentration in the blood, due to fasting, physical activity, or a targeted diet, is a natural physiological condition. Ketosis is characterized by the presence of ketone bodies in the blood, with concentrations that increase from 0.1 mmol/L to about 5–8 mmol/L, a value that remains stable over time when the intake of carbohydrates is kept below certain levels [40,79]. The ketogenic diet should not be confused with pathological situations, such as metabolic ketoacidosis. This diet is safe when performed under the supervision of a trained professional and has negligible side effects in the short to medium term. Although the ketogenic diet has been used to successfully treat migraine sufferers as early as 1928, only in recent years has this strategy returned to the forefront, first with individual case studies, then with clinical studies. The ketogenic diet has been shown to be effective both in individual subjects and during clinical trials, with a reduction in the frequency and intensity of attacks, reduced use of drugs, and in some cases, the disappearance of migraines. The ketogenic diet can contribute to restoring brain excitability and metabolism and counteracting neuroinflammation in migraine, although its precise mechanism is still unknown [80]. It is not yet clear how physiological ketosis can provide these positive effects [81]. Speculation on the molecular mechanisms related to a ketogenic diet has been recently reported [82]. Migraine is a complex disorder in which the balance between the activation and inhibition of certain areas of the cerebral cortex is altered. This includes changes in blood flow to these areas and the involvement of the trigeminal nerve and other brain structures, which are responsible for the symptoms that characterize the attack. According to recent studies, migraines could be due to an energy deficit in the brain, which occurs when the affected tissues are subjected to strong oxidative stress or metabolic processes are not sufficient to cope with the high energy needs of neurons. Ketone bodies produced in the liver during ketosis, particularly β-hydroxybutyrate, can cross the blood-brain barrier and reach neurons, which use ketone bodies instead of glucose to produce energy with great efficiency [83]. Because of the ketone bodies, the energy produced by the neurons’ mitochondria increases, and the production of free radicals is reduced, causing a significant improvement in metabolic processes. This could compensate for a preexisting deficit. Ketone bodies can promote the degradation of glutamate, an important cerebral excitatory mediator, and therefore reduce the excitability of the cortex. They can also protect the cortex from neuroinflammatory processes, contributing to a significant reduction of some important inflammatory mediators such as TNF-α and NFκB. Inflammation is an important component of migraine, which contributes to the activation of the fibers of the trigeminal nerve that are responsible for the sensation of pain [84,85]. The ketogenic diet, due to its particular composition, which involves a reduced intake of fiber, can cause significant alterations in the intestinal microbiota. Several studies have shown an improvement in the bacterial composition, marked by increased levels of Bacteroidetes and Prevotella. These changes could lead to positive effects on migraine progression through mechanisms involving bacterial metabolites and neuropeptides that are yet to be identified [86]. During a ketogenic diet, the patient cannot consume cereals or products based on cereals, legumes, tubers, fruit, or any foods that contain significant quantities of sugars or starches. Total sugar intake should be reduced to below 30 g per day, protein intake should typically be approximately 1.4 g per kg of body weight, and any remaining calories should be derived from high-quality fat. The patient can consume meat, fish, eggs, dried oily fruit, and vegetables, with a caloric intake that must be calculated according to the needs and objectives of the subject [87]. Hydration is very important because excess ketone bodies are eliminated in the urine, and it is necessary to maintain sufficient intake of fluids. In most cases, one or two months of a ketogenic diet is enough to reduce migraine attacks. Then, patients can gradually switch to a low-glycemic index diet, in which it is possible to consume whole grains, legumes, and fruit while avoiding significant glycemic peaks. The beneficial effects of the ketogenic diet can be maintained for several months, and when they begin to subside, it is possible to resume the diet. This can be achieved by alternating the two diets in successive cycles, employing methods and times that allow better disease management. Targeted supplements may be needed during a ketogenic diet. It should be noted that the ketogenic diet is not suitable for everyone; there are important contraindications, including type I diabetes, pregnancy, and breastfeeding. Moreover, the ketogenic diet is not a do-it-yourself diet and must not be managed directly by the patient without the intervention of specialized personnel [88]. Many studies associate migraines with increased insulin levels. The hypothetical relationship between obesity and headache has been linked to a high release of inflammatory markers. Among the studied proinflammatory agents, an elevated level of C-reactive protein (CRP), known as a marker of systemic inflammation, has been reported in both obese individuals and patients with migraine [89,90]. Serotonin is responsible for food consumption and body weight regulation, and these processes are controlled by the hypothalamus. During a migraine attack, the concentrations of this neurotransmitter increase significantly; it is released in large quantities by the platelets, resulting in vasoconstriction of the arteries and arterioles and, consequently, pain [91]. Another appetite regulator that could contribute to migraines is orexin A. An increase in the level of orexin A in cerebrospinal fluid has been observed in migraine sufferers [92]. Orexin A could have antinociceptive characteristics and might play a role in the compensatory reaction to pain and contribute to the perception of hunger. Five studies in the literature have addressed the effect of low-fat diets as a means of migraine/headache prophylaxis. In 1999, a study was conducted to evaluate the role of the low-fat diet for migraine control in 54 adults [93]. Patients were instructed to limit their fat intake to less than 20 g/day for 12 weeks. At the end of the trial, the patients reported a significant reduction in the frequency and intensity of headaches and the need for drug treatments. In another cross-study of 63 adults with episodic or chronic migraine, a low-fat diet (<20% of total daily energy consumption) for 3 months significantly reduced the frequency and severity of headache attacks. In this study, the participants did not reduce their total fat intake to less than 45 g/d and used olive oil as the main source of fat intake [94]. Furthermore, based on the theory of the probable effects of different types of fat on the characteristics of headache, a randomized study evaluated the effect of the intake of omega-3 and omega-6. Fifty-five adults with chronic migraine reduced their intake of omega-6 fats or reduced omega-6 fats along with an increased consumption of omega-3. After each week, individuals taking high omega-3 levels in combination with a low-omega-6 diet showed greater headache improvement than patients with an omega-6-reduced diet. The amount and type of fat intake influences inflammatory responses. The balance between omega-6 and omega-3, two main fatty acids that compete with arachidic acid as a precursor of eicosanoid biosynthesis, contributes to inflammatory control in response to environmental metabolic changes. Prostaglandins, which are made up of essential fatty acids, contribute to platelet function and the regulation of vascular tone. It is generally believed that a high-fat diet raises plasma LDL cholesterol and, consequently, increases platelet aggregation. The increase in platelet aggregation is a crucial factor contributing to an increase predisposition to headaches. Each migraine patient can have a specific trigger or a unique set of triggers. It is known that some types of foods and drinks can act as triggers. Cheese, chocolate, citrus fruits, alcohol, coffee, tomatoes, carbohydrates, leavened products, and red wine are among the proposed foods that can trigger migraine attacks. However, there is no consensus among the identification of food triggers in headache. For example, as previously mentioned, chocolate has been introduced as one of the triggers of headaches [95]. In a 1997 double-blind study by Marcus et al., the effect of chocolate was compared to that of carob on 63 female subjects with chronic headaches, producing different results. The study was conducted following the prescription of a diet in which vasoactive amine-rich foods were restricted for 2 weeks. However, after the administration of chocolate and carob (both in two samples), there was no difference in the positive effects of these agents on headache [96].

## 7. Influence of Migraine on Lifestyle

Several studies conducted on migraine have focused on the negative impact of migraine attacks on the patient’s work, home, and social environment, also known as “quality of life” [97]. It is recommended to use drugs for the treatment of migraine attacks only when healthy lifestyle behaviors are not enough to reduce migraine attacks. These behaviors include finding a way to talk about migraine pain and ask for help if needed (silence about pain can lead to miscommunication), eat regular meals, and avoid skipping or delaying meals. In addition, maintaining adequate hydration with noncaffeinated beverages, ensuring proper sleep, maintaining regular sleep hours, reducing stress by utilizing relaxation strategies and other stress management techniques, maintaining regular exercise, and avoiding migraine triggers, may be recommended [98]. In Italy, about 300 patients with migraine were examined in a study conducted at a headache center [99]. The mean values obtained in this survey indicated that the ability to perform household chores and personal and social activities was affected by migraine in 14.5 days/3 months. Over the same period, total or partial migraine attacks affected the work activities of patients for an average of 8.8 days/3 months, including 2.2 days of absenteeism and 6.6 days of attendance. Almost all interviewed subjects affected by migraine claim to have difficulty in performing normal daily activities, leading them to make sacrifices in their social and professional lives. Regular physical activity is generally associated with lower migraine prevalence [100,101]. A recent cross-sectional analysis of the Brazilian Longitudinal Study of Adult Health [102] evidenced that meeting the World Health Organization (WHO) physical activity guidelines for physical activity levels in the leisure time is associated with lower migraine occurrence. Moderate physical activity levels in leisure time tends to reduce migraine with aura, whereas vigorous physical activity levels in leisure time tends to reduce migraine without aura. However, during the COVID-19 lockdown, significant changes in physical activity and working habits of people with migraine were observed. Despite the lower physical activity, improved migraine-related symptoms were evidenced, which could depend on different lifestyle habits [103,104]. Moreover, the practice of activities such as yoga, diaphragmatic breathing, chromotherapy courses, and music therapy may represent useful aids in the prevention of migraines [105,106,107,108,109,110]. Finally, comorbid migraine may be relatively common amongst chronic rhinosinusitis patients, and its presence is associated with significantly worse quality of life. Dizziness as a symptom in chronic rhinosinusitis patients may be particularly indicative of migraine [111].

## 8. Migraine and Food

Research has revealed the presence of surprising connections between the various types of migraine and food. Some foods can cause migraine attacks, whereas others can prevent and even cure them. Coffee, for example, can sometimes ward off a migraine crisis, and foods rich in magnesium, calcium, complex carbohydrates, and fiber have been used to treat this disease [112,113]. Some reports suggest that *Zingiber officinale* (ginger)—the common cooking spice—may aid in the prevention and treatment of migraines without leading to any of the side effects of medications [114,115]. Feverfew herb has also been shown to be effective in the management of migraines in placebo-controlled studies. These studies also indicate *Cannabis sativa* (cannabis), intranasal *Capsicum annuum* (cayenne), and *Lavandula stoechas* (Spanish lavender) volatile oil for treatment and prevention. Moreover, *Petasites hybridus* (butterbur) root, *Curcuma longa* (turmeric) and fish oil, *Citrus medica* (citron) fruit, *Tanacetum parthenium* (feverfew), *Tanacetum parthenium* (feverfew) and *Salix alba* (white willow), *Ginkgo biloba* (ginkgo), and *Lippia alba* (bushy matgrass), have been studied for migraine prevention, although the latter three have little published evidence of efficacy [116]. In 1983, researchers from the Hospital for Sick Children in London reported the results of their observations on 88 children with severe and frequent migraine crises who had started an elimination diet. Of these 88 children, 78 recovered completely and 4 improved significantly [117]. In the same study, some children who also had seizures noticed that they no longer experienced seizure episodes. Researchers then began reintroducing various foods into the diet and found that these triggered the resumption of migraine attacks in all but 8 of the children. In subsequent trials using disguised foods, most of the children became asymptomatic again when the foods that triggered the seizures were avoided. Migraine crises recurred when offending foods were added to their diets. In adults, between 20 and 50% of patients experience the reduction or disappearance of headaches when common trigger foods are eliminated from the diet. There are also harmless foods that never contribute to headaches or other painful conditions [77]. These foods include rice (especially whole grain), cooked green vegetables, such as broccoli, spinach, chard, and kale, cooked orange-colored vegetables, such as carrots and sweet potatoes, cooked yellow-colored vegetables, such as pumpkin, cooked or raw fruit, such as cherries, blueberries, pears, and plums (but not citrus fruits, apples, bananas, peaches, or tomatoes), and water (still, or with added carbon dioxide, such as Perrier); other drinks, even herbal teas, can be triggers. Flavoring substances such as modest amounts of salt, maple syrup, and vanilla extracts are generally well-tolerated. Common trigger foods often provoke a crisis in predisposed individuals. Just as some reactions of intolerance to food are manifested by skin rashes, migraine patients have a reaction in the blood vessels and nerves. The most common migraine trigger foods are represented by dairy products, chocolate, eggs, citrus fruits, meat, wheat (bread, pasta, etc.), nuts and hazelnuts, tomatoes, onions, corn, apples, and bananas. Also counted among the worst triggers are alcoholic beverages (especially red wine), beverages containing caffeine (coffee, tea, and Coca-Cola), monosodium glutamate, aspartame (a sweetener), and nitrites. Foods that are not on the previous two lists should be considered possible but unlikely trigger foods. Almost every common food not included in the aforementioned list (pain-safe) has triggered the onset of migraine crises in isolated subjects in clinical research studies; therefore, these foods cannot be considered completely above suspicion. Dietary Approaches to Stop Hypertension (DASH) have indicated that lower amounts of sodium (< 2400 mg/day), as well as higher amounts of magnesium, potassium, and calcium, are effective in reducing the frequency, duration, and severity of migraine headaches in adult patients [118,119,120]. Moreover, adherence to the Mediterranean dietary pattern is generally associated with lower headache and migraine frequency and duration [121]. The human gut microbiome, considered the second genome and brain of human body, is thought to be closely related to migraine [122]. Patients with irritable bowel syndrome (IBS) and inflammatory bowel disease (IBD), which are severe gut disorders associated with gut permeability and inflammation, are more likely to have migraines [123]. Thus, the gut microbiome is impaired in patients with migraine, and several studies have been conducted to identify the benefits of pre- and probiotic use for migraine. In an uncontrolled observational study on 1020 patients, researchers found that multispecies probiotic formulations can reduce the intensity and the frequency of migraine attacks [124]. However, another randomized placebo-controlled study conducted on 63 patients demonstrated that the use of multispecies probiotics did not significantly affect intestinal permeability or inflammation in comparison with patients treated with placebo [125]. Moreover, recent findings highlight that dietary triggers exist for migraine, for example, coffee and alcohol [126]. The role of caffeine in migraine headaches is ambiguous, from trigger to treatment. A recent review by Nowaczewska et al. (2020) [127] summarized studies concerning the prevalence of caffeine/caffeine withdrawal as a migraine trigger (21 studies) and other studies evaluating caffeine in acute migraine treatment (7 studies). Among these, in 17 studies, caffeine/caffeine withdrawal was found to be a migraine trigger in a small percentage of participants (ranging from 2% to 30%), whereas all treatment studies found caffeine to be safe and effective in acute migraine treatment, mostly in combination with other analgesics, suggesting that there is insufficient evidence to recommend caffeine cessation to all migraine patients. However, it should be noted that caffeine overuse may lead to migraine chronification, and sudden caffeine withdrawal may trigger migraine attacks. Migraine sufferers should not exceed 200 mg daily of caffeine consumption. Yuan et al. (2022) [128] conducted a Mendelian randomization study to evaluate if alcohol and coffee consumption and smoking are causally associated with risk of developing migraine. Data suggest that causal evidence exists in a protective role of moderate coffee consumption and a detrimental role of cigarette smoking in migraine. Drinking alcohol has been associated with an enhanced risk of tension-type headaches and migraine [129]. However, recently published studies have not confirmed this relationship. The existing literature is inconclusive; however, migraine patients avoid alcohol. Recently, Błaszczyk et al. (2023) [130] suggested that alcohol consumption and migraine are inversely correlated. The exact mechanism behind this observation may indicate that migraine leads to alcohol-avoidance, rather than alcohol having any protective role against migraine. No relationship was found between tension-type headaches and drinking. 

## 9. Conclusions

This review is focused on the role of CGRP in migraine pathogenesis, along with the corresponding drugs that target the receptor. Additionally, it considers the influence of triggers and physical activity in the management of migraine. Moreover, the importance of a low-carb diet in the management of migraine has been underlined. The antagonists of the CGRP receptor are very effective drugs in the treatment of migraine, given their high diffusion in the brain. In fact, a correlation emerged between the analysis of the physiopathological and pharmacological aspects of migraine, extending to a proper dietary approach with specific nutritional interventions aimed at modulating symptoms. Several studies have shown that migraine is related to endothelium-dependent vasodilation caused by the release of endogenous mediators. Because of the discovery of the role of CGRP, a significant reduction in monthly migraine days may be observed. Gepants showed side effects such as liver toxicity, whereas monoclonal antibodies, in addition to efficacy, show safety and tolerability. Monoclonal antibodies that target CGRP and its receptor have been shown to modify and improve the patient’s quality of life and decrease migraine-related disability. Monoclonal antibody therapy reduces the frequency, duration, and intensity of migraine attacks, as well as the use of other acute, ineffective, or poorly tolerated medications, thereby preventing the onset of drug abuse migraines. The advantage of a long half-life allows for monthly or less frequent administration, improving adherence to therapy. This type of therapeutic approach allows patients to manage their disease by improving their sense of personal control. Antibody therapy has been shown to be effective in reducing both episodic and chronic migraine attacks starting from the first three months, improving the quality of life related to the disease, and decreasing the stress and psychological symptoms associated with headaches. The introduction of migraine-specific preventative therapies based on solid science is growing now. In addition to the use of drugs for the reduction of migraine symptoms, possible improvement can be obtained by eliminating the triggers. For example, there foods such as caffeine, cured meats, aged cheeses, dried fruit should be considered. Other factors, such as environmental, hormonal, physical, and emotional factors, can also contribute to the development of migraines. Following a specific lifestyle, for example following a balanced diet, practicing sports such as yoga and respiratory gymnastics, taking chromotherapy courses, practicing homeopathy, and attending music therapy, is a nonpharmacological treatment that is useful in the prevention of migraines. Therefore, nutrition and lifestyle can play a preventive role together with preventive drugs, such as calcium antagonists, beta-blockers, antiepileptics, and antidepressants, in the management of migraine.

## 10. Perspectives

The validity and importance of CGRP as a therapeutic target and the development of monoclonal antibodies targeting the peptide represent an extraordinary opportunity for antimigraine therapy. The certainty that CGRP has a fundamental role in migraine has allowed for a broader understanding of the disease and laid the foundation for further studies that could lead to other new therapies. Anti-CGRP monoclonal antibodies constitute the first class of preventive therapy based on a targeted and distinctive mechanism of the disease. Moreover, providing tools to train and educate people who suffer from migraines can reduce the impact that the disease has on daily life. Being informed about the various aspects of this pathology allows the patient to be able to manage the migraine and efficiently exploit the available resources, thus improving the quality of life and restoring the normal course of work and daily activities.

## Figures and Tables

**Table 1:** Drugs used for migraine therapy.

Structure	Name	Class
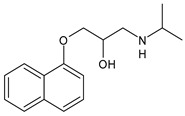	Propranolol	β-blocker
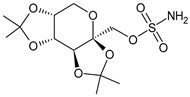	Topiramate	β-blocker
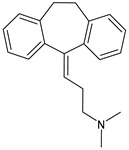	Amitriptyline	Antidepressant
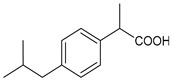	Ibuprofen	FANS
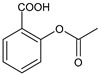	Acetylsalicylic acid	FANS
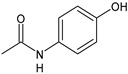	Acetaminophen	FANS
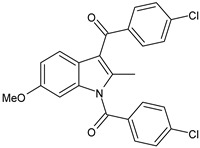	Indomethacin	FANS
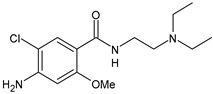	Metoclopramide	Anti-emetic
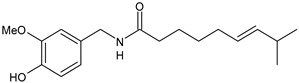	Capsaicin	Analgesic
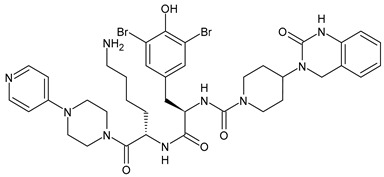	Olcegepant	CGRP inhibitor
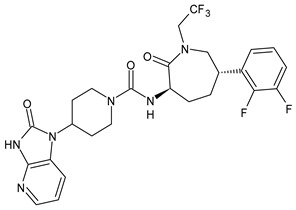	Telcagepant	CGRP inhibitor
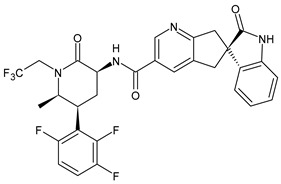	Atogepant	CGRP inhibitor
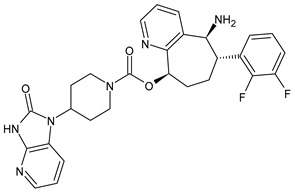	Rimegepant	CGRP inhibitor
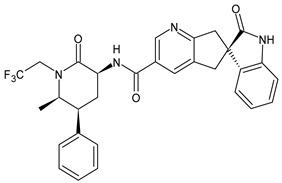	Ubrogepant	CGRP inhibitor

## Data Availability

Supporting data are available within the manuscript.
